# Evaluation of the prognostic value of the anatomical characteristics of the bony structures in the shoulder in bursal-sided partial-thickness rotator cuff tears

**DOI:** 10.3389/fpubh.2023.1189003

**Published:** 2023-05-26

**Authors:** Jun Liu, Simin Dai, Hui Deng, Dewei Qiu, Li Liu, Mingzhang Li, Zhijun Chen, Jiawei Kang, Jun Tao

**Affiliations:** ^1^Department of Orthopaedic Surgery, The Second Affiliated Hospital of Nanchang University, Nanchang, Jiangxi, China; ^2^Department of Emergency, The Second Affiliated Hospital of Nanchang University, Nanchang, Jiangxi, China; ^3^Department of Gastrointestinal Surgery, The First Affiliated Hospital of Nanchang University, Nanchang, Jiangxi, China

**Keywords:** rotator cuff tears, scapula anatomy, greater tuberosity angle, acromion index, bursal-sided partial-thickness rotator cuff tears, shoulder characteristics

## Abstract

**Background:**

In recent studies, individual scapular anatomy has been found to be related to degenerative full-thickness rotator cuff tears. However, research on the relationship between the anatomical characteristics of shoulder radiographs and bursal-sided partial-thickness rotator cuff tears (PTRCTs) is limited, and the risk factors for this pathology still need to be determined.

**Methods:**

The bursal-sided PTRCTs group included 102 patients without a history of shoulder trauma who underwent arthroscopy between January 2021 and October 2022. A total of 102 demographically matched outpatients with intact rotator cuffs were selected as the control group. Radiographs were used to measure the lateral acromial angle (LAA), critical shoulder angle (CSA), greater tuberosity angle (GTA), β-angle, acromion index (AI), acromiohumeral distance (AHD), acromial tilt (AT), acromial slope (AS), acromial type, and acromial spur by two independent observers. Multivariate analyses of these data were used to identify potential risk factors for bursal-sided PTRCTs. Receiver operating characteristic (ROC) analysis was performed to assess the sensitivity and specificity of CSA, GTA, and AI for this type of pathology.

**Result:**

The β-angle, AHD, AS and acromion type showed no difference between bursal-sided PTRCTs and controls (*p* = 0.009, 0.200, 0.747 and 0.078, respectively). CSA, GTA and AI were significantly higher in bursal-sided PTRCTs (*p* < 0.001). LAA, β-angle and AT were significantly lower in bursal-sided PTRCTs. Multivariate logistic regression analysis demonstrated significant correlations between the acromial spur (*p* = 0.024), GTA (*p* = 0.004), CSA (*p* = 0.003) and AI (*p* = 0.048) and bursal-sided PTRCTs. The areas under the ROC curves for AI, CSA, and GTA were 0.655 (95% CI 0.580–0.729), 0.714 (95% CI 0.644–0.784), and 0.695 (95% CI 0.622–0.767), respectively.

**Conclusion:**

Acromial spur, GTA, CSA, and AI were independent risk factors for bursal-sided PTRCTs. Furthermore, CSA was the most powerful predictor of bursal-sided PTRCTs compared to GTA and AI.

## Background

Rotator cuff tears (RCTs) are a frequent cause of shoulder pain and functional limitations (1), and their incidence increases with age. Teunis et al. (2) found that the prevalence of RCTs was 30% in individuals over 60 years of age and up to 62% in individuals over 80 years of age.

There is currently a lack of understanding regarding the pathogenesis of RCTs, and some research (3–5) suggests that it may be related to the heterogeneity in the anatomical characteristics of the individual shoulder joint’s bony structures. Compared with full-thickness tears, the incidence of partial-thickness rotator cuff tears (PTRCTs) is reported to be higher (6–8), and the majority of full-thickness tears are caused by PTRCTs (9). PTRCTs can be on the articular side of the tendon, on the bursal side of the tendon, or intratendinous (8, 10). Because the anatomic location, biomechanics, and vascular supply conditions of PTRCTs vary, so do the causes of their formation. Most studies have focused on articular-sided and intratendinous PTRCTs (11–13). Few studies have evaluated bursal-sided PTRCTs, and the risk factors for this pathology still need to be determined.

Several anatomical characteristics of the bony structures in the shoulder, such as acromion type (3), critical shoulder angle (CSA) (5), and acromion index (AI) (4), have been reported to be correlated with full-thickness tears (14–16). However, due to the uniqueness of bursal-sided PTRCTs, their association with the anatomical characteristics of the shoulder remains unknown. Therefore, this study mainly focused on the relationship between the anatomical characteristics of the shoulder and bursal-sided PTRCTs.

## Patients and methods

### Patients

After obtaining ethical approval (Review [2019] No. 115), we retrospectively identified 204 patients of our institution between January 2021 and October 2022 to be included in this study. They gave informed consent and, after examination, were divided into two groups. The bursal-sided PTRCTs group consisted of 102 consecutive patients who underwent arthroscopically confirmed isolated bursal-sided PTRCTs. These patients underwent arthroscopy after at least 3 months of ineffective conservative treatment such as nonsteroidal anti-inflammatory drugs, physical therapy, functional exercises, etc. Only patients with available preoperative true anteroposterior (17) and standardized supraspinatus outlet view (18) were included. Patients with inflammatory disease, a history of trauma or previous surgery, scapula or greater tuberosity fractures, and glenohumeral arthritis of the affected shoulder were excluded. Based on the demographics of the bursal-sided PTRCTs, an age and sex-matched control group was formed. These subjects were retrieved from a data registry of a consecutive series of patients treated for shoulder pain or limitation in range of motion. In all of these patients, the integrity of the rotator cuff was confirmed by a detailed physical examination and magnetic resonance imaging (MRI). Similar to bursal-sided PTRCTs, only patients with available preoperative true anteroposterior and standardized supraspinatus outlet view were included. In contrast, individuals with previous surgery or dislocation, scapula or greater tuberosity fractures, and glenohumeral arthritis of the affected shoulder were excluded.

### Radiologic assessment

For the genuine anteroposterior radiograph (17), the patient’s scapula was positioned next to the X-ray cassette. The arm was held neutral, with the elbow extended and the thumb pointed forward. Beam alignment was caudal by 20°. For the outlet-view radiograph (18), the symptomatic shoulder was rotated 30 degrees away from the X-ray stand with the arm hanging. Beam alignment was 10–15°caudo-cranial and tangent to the scapula. Acromion type and spur, acromial tilt (AT), and acromial slope (AS) were measured on outlet view. Lateral acromial angle (LAA), acromiohumeral distance (AHD), greater tuberosity angle (GTA), AI, CSA, and β-angle on anteroposterior radiographs. Two independent observers performed parameters measurements at 2 different times separated by 1 months, each measurement taken as the average between the two observers. At both assessments they were blinded to the group identity of the radiographs.

### Lateral acromial angle

**Figure 1:**
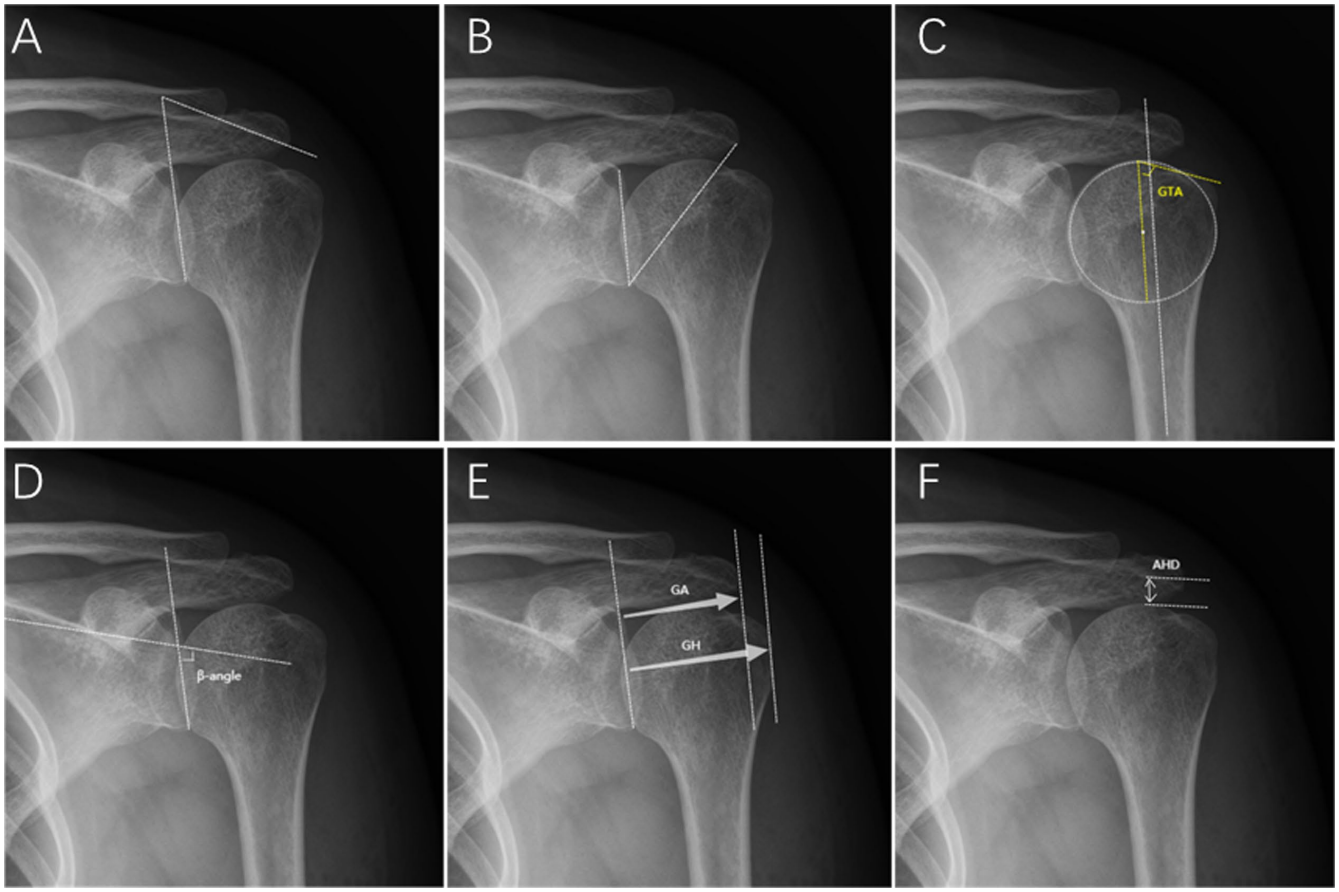
Radiographic measurements on the true anteroposterior radiograph. **(A)** Lateral acromial angle (LAA). **(B)** Critical shoulder angle (CSA). **(C)** Greater tuberosity angle (GTA). **(D)** β-angle. **(E)** Acromion index (AI): GA/GH. **(F)** Acromiohumeral distance (AHD).

According to Banas et al. (19), LAA is the angle between the line connecting the upper and lower edges of the scapular glenoid and the extension of the lower surface of the acromion (Figure 1A).

### Critical shoulder angle

As reported by Moor et al. (5), CSA is the angle between the line passing through the superior and inferior points of the scapular glenoid and the line that passes through the inferior point of the glenoid and the most lateral point of the acromion (Figure 1B).

### Greater tuberosity angle

As reported by Cunningham et al. (20), The GTA consists of the angle between a parallel line to the diaphyseal axis that passes through the humeral head center of rotation and a line that connects the superior border of the humeral head to the superolateral edge of the greater tuberosity (Figure 1C).

#### βangle

According to Maurer et al. (21), β-angle is the angle between the floor of the supraspinatus fossa and the glenoid fossa line (Figure 1).

#### Acromial tilt

**Figure 2:**
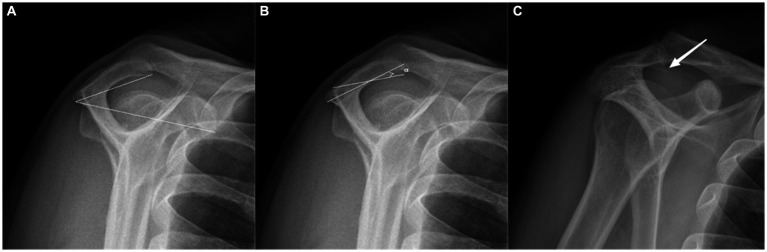
Radiographic measurements on the outlet-view radiograph. **(A)** Acromial tilt (AT), a type II (curved) acromion according to the Bigliani. **(B)** Acromial slope (AS). **(C)** Acromial spur.

As described by Kitay et al. (22) and Aoki et al. (23), this angle is determined by connecting with two lines the most posteroinferior point of the acromion to the inferior tip of the coracoid and to the anteroinferior acromion (Figure 2A).

#### Acromion index

According to Nyffeler et al. (4), AI is the ratio of the distance from the scapular glenoid to the most lateral aspect of the acromion to the distance from the scapular glenoid to the most lateral aspect of the humeral head (Figure 1E).

#### Acromiohumeral distance

Golding et al. (24) described that AHD is the distance between the subacromial surface and the humeral head, representing the subacromial space’s volume (Figure 1F).

#### Acromial type

The acromial type was classified according to Bigliani et al. (3). Type I is flat, type II a curved, and type III is a hooked undersurface of the acromion (Figure 2A).

#### Acromial slope

As described by Biglian et al. (3), the most anterior and posterior points on the inferior acromion are marked. The midway point on the inferior acromion is identified, and lines are drawn from it to the already marked anterior and posterior acromion points. The AS is the complement of the resulting angle measured (Figure 2B).

#### Acromion spur

Ogawa et al. (25) described that an acromion spur is a bony convex on the subacromial surface that often leads to impingement syndrome (Figure 2C).

### Statistics

The SPSS for Windows program (v25; IBM SPSS Statistics, United States) was used for statistical analysis. Continuous variables were expressed as mean ± standard deviation, and categorical variables were expressed as n (%). Shapiro–wilk was used to test whether the data were in accordance with the normal distribution, in which age, CSA, βangle, and AT were in accordance with the normal distribution data, so two independent samples t-test was used. The Mann–Whitney U-test with two independent samples was used for the variables such as LAA, AHD, AS, AI, GTA, and body mass index (BMI), which did not conform to the normal distribution. Categorical variables were tested by the Chi-Square test or Fisher’s exact test. The multivariate logistic regression was used to analyze the parameters related to bursal-sided PTRCT. Inter- and intra-observer measurement reliability was assessed on intraclass correlation coefficient (ICC) (26) and kappa statistics (27) for the continuous and categorical variables respectively, using the absolute agreement definition in a two-way mixed effect. The Pearson correlation coefficient (PCC) was used to calculate correlations between parameters, which were graded as excellent (0.81–1.00), good (0.61–0.80), moderate (0.41–0.60), fair (0.21–0.40), or poor (0.00–0.20) (28). Receiver operating characteristic (ROC) curve analyses were used to determine the cut-off values and to assess the sensitivity and specificity for significant parameters. *p* < 0.05 was considered statistically significant.

## Result

### General data

All demographic data of the patients are listed in Table 1. In the bursal-sided PTRCTs group, there were 60 (58.8%) women and 42 (41.2%) men; the mean age was 56.4 ± 12.6 years; 32 left shoulders and 70 right shoulders; the average BMI was 23.2 ± 2.7; Of these patients, 43 (42.2%)were smoking, and 59 (57.8%)were non-smoking. In control group, There were 54 (52.9%)women and 48 (47.1%)men; the mean age was 53.6 ± 13.7 years; 41 left shoulders and 61 right shoulders; the average BMI was 23.3 ± 2.7; Of these patients, 30 (29.4%)were smoking and 72 (70.6%)non-smoking. There was no significant difference between the two groups in these basic demographic data (*p* > 0.05; Table 1). Characteristics for the true anteroposterior radiograph.

**Table 1:** Demographic date of bursal-sided partial-thickness rotator cuff tears and control group.

	Bursal-sided	Control	*p* value
Gender [*n* (%)]			
Male	42 (41.2)	48 (47.1)	0.481^a^
Female	60 (58.8)	54 (52.9)	
Age (years)	56.4 ± 12.6	53.6 ± 13.7	0.124^b^
Limb [n (%)]			
Left	32 (31.4)	41 (40.2)	0.243^a^
Right	70 (68.6)	61 (59.8)	
Body mass index (kg/m^2^)	23.2 ± 2.7	23.3 ± 2.7	0.456^c^
Smoking [n (%)]			
Yes	43 (42.2)	30 (29.4)	0.079^d^
No	59 (57.8)	72 (70.6)	

a Chi-squared test.

b *t*-test.

c Mann–Whitney *U*-test.

d Fisher’s exact test.

For parameters reflecting a lateral extension of the acromion, the mean LAA values were significantly lower in bursal-sided PTRCTs than in the control group (76.6° ± 9.0° vs. 81.1° ± 10.2°, *p* < 0.001). However, the mean CSA was significantly higher in bursal-sided PTRCTs than in the controls (36.8° ± 6.7° vs. 31.4° ± 7.3°, *p <* 0.001). Similarly, the mean AI was significantly higher in bursal-sided PTRCTs than in the controls (0.73 ± 0.10 vs. 0.67 ± 0.11, *p* < 0.001). For parameters reflecting glenoid inclination, the average values of the β-angle were significantly lower in bursal-sided PTRCTs than in the control group (71.0° ± 8.9° vs. 74.2° ± 8.4°, *p* = 0.009). Besides, we found the average GTA of bursal-sided PTRCTs was significantly higher than that of the control group (71.7° ± 8.8° vs. 68.1° ± 6.0°, *p* < 0.001). Finally, the mean AHD had no significant difference between the two groups (0.73 ± 0.2 vs. 0.78 ± 0.2, *p* = 0.200; Table 2**).**

**Table 2:** Comparison of bursal-sided partial-thickness rotator cuff tears and control group.

Variable	Bursal-sided	Control	*p* value
LAA (°)	76.6 ± 9.0	81.1 ± 10.2	<0.001^c^
CSA (°)	36.8 ± 6.7	31.4 ± 7.3	<0.001^b^
GTA (°)	71.7 ± 8.8	68.1 ± 6.0	<0.001^c^
β-angle (°)	71.0 ± 8.9	74.2 ± 8.4	0.009^b^
AI	0.73 ± 0.10	0.67 ± 0.11	<0.001^c^
AHD (mm)	0.73 ± 0.2	0.78 ± 0.2	0.200^c^
AT (°)	30.7 ± 6.4	33.3 ± 7.0	0.006^b^
AS (°)	30.2 ± 8.0	29.6 ± 6.7	0.747^c^
Acromion type [*n* (%)]			
I	32 (31.4)	44 (43.1)	0.078^a^
II	53 (52.0)	50 (49.0)	
III	17 (16.7)	8 (7.8)	
Acromial spur [*n* (%)]			
yes	15 (14.7)	4 (3.9)	0.014^a^
no	87 (85.3)	98 (96.1)	

a Chi-squared test.

b *t*-test.

c Mann–Whitney U-test.

d Fisher’s exact test.

LAA, lateral acromial angle; AHD, acromiohumeral distance; AS, acromial slope; GTA, greater tuberosity angle; AI, acromion index; CSA, critical shoulder angle; AT, acromial tilt.

### Characteristics for the outlet-view radiographs

For parameters that reflect acromion, we found the mean AT values were significantly higher in bursal-sided PTRCTs than in the control group (33.3° ± 7.0° vs. 30.7° ± 6.4°, *p* = 0.006). Additionally, the acromial spur in the bursal-sided PTRCTs group is more common than the control group [15 (14.7%)vs. 4(3.9%), *p* = 0.014]. For AS, found no difference between groups (30.2° ± 8.0° vs. 29.6° ± 6.7°, *p* = 0.747). Lastly, the most common type of acromion between the two groups remained type II. There was no relationship between acromial morphology and bursal-sided PTRCTs (*p* = 0.078; Table 2).

### Multivariate logistic regression analysis

**Figure 3:**
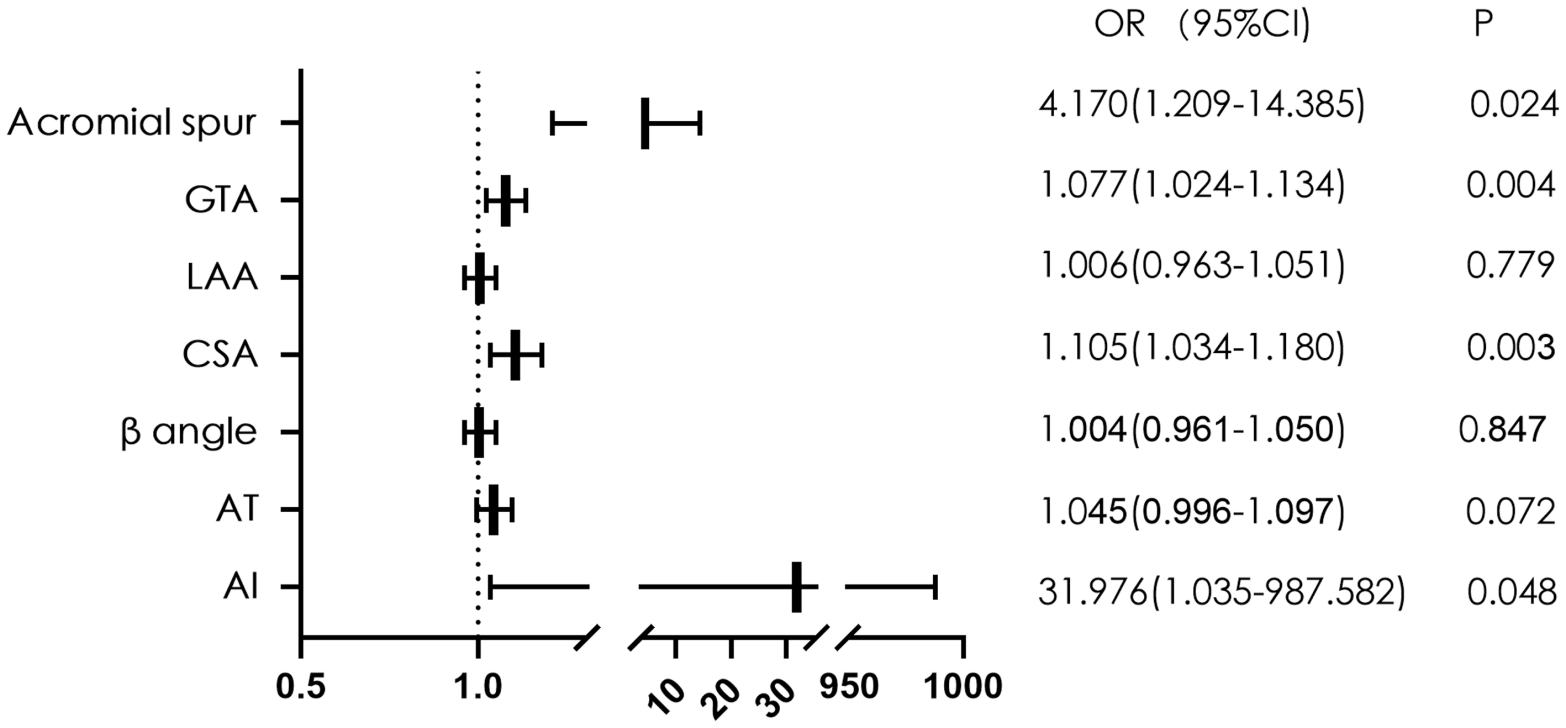
The forest plot of multivariate logistic regression analysis of factors related to bursal-sided partial-thickness rotator cuff tears. GTA, greater tuberosity angle; LAA, lateral acromial angle; CSA, critical shoulder angle; AT, acromial tilt; AI, acromial index; OR, odds ratio.

We further performed a multivariate logistic regression analysis for the variables that were significant differences between the two groups. The results showed that the acromial spur (OR 4.170, 95%CI 1.209–14.385), GTA (OR 1.077, 95%CI 1.024–1.134), CSA (OR 1.105, 95%CI 1.034–1.180) and AI (OR 31.976, 95%CI 1.035–987.582) were significantly associated with bursal-sided PTRCTs (*p* < 0.05; Figure 3).

### Correlation and ROC curve analysis

**Figure 4:**
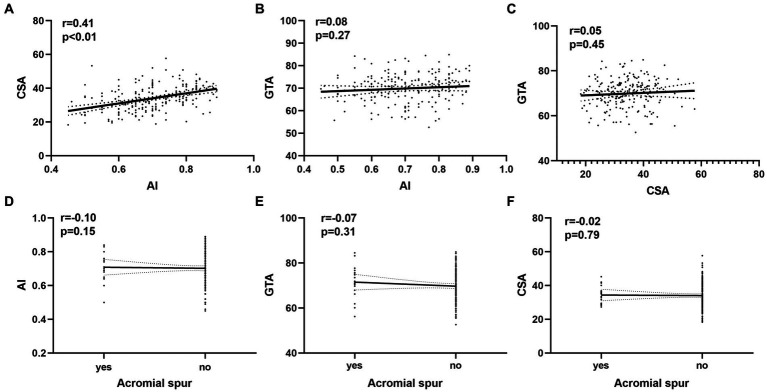
The relationship between variables and the bursal-sided partial thickness of rotator cuff tears. **(A)** The correlations between AI and CSA. **(B)** The correlations between GTA and AI. **(C)** The correlations between GTA and CSA. **(D)** The correlations between AI and acromial spur. **(E)** The correlations between GTA and acromial spur. **(F)** The correlations between CSA and acromial spur. AI, acromial index; CSA, critical shoulder angle; GTA, tuberosity angle;

We found a moderate correlation between CSA and AI (r = 0.41; *p* < 0.01). However, Poor correlations were found between GTA and AI and CSA, acromial spur and the other parameters (r < 0.2; *p* > 0.05; Figure 4).

**Figure 5:**
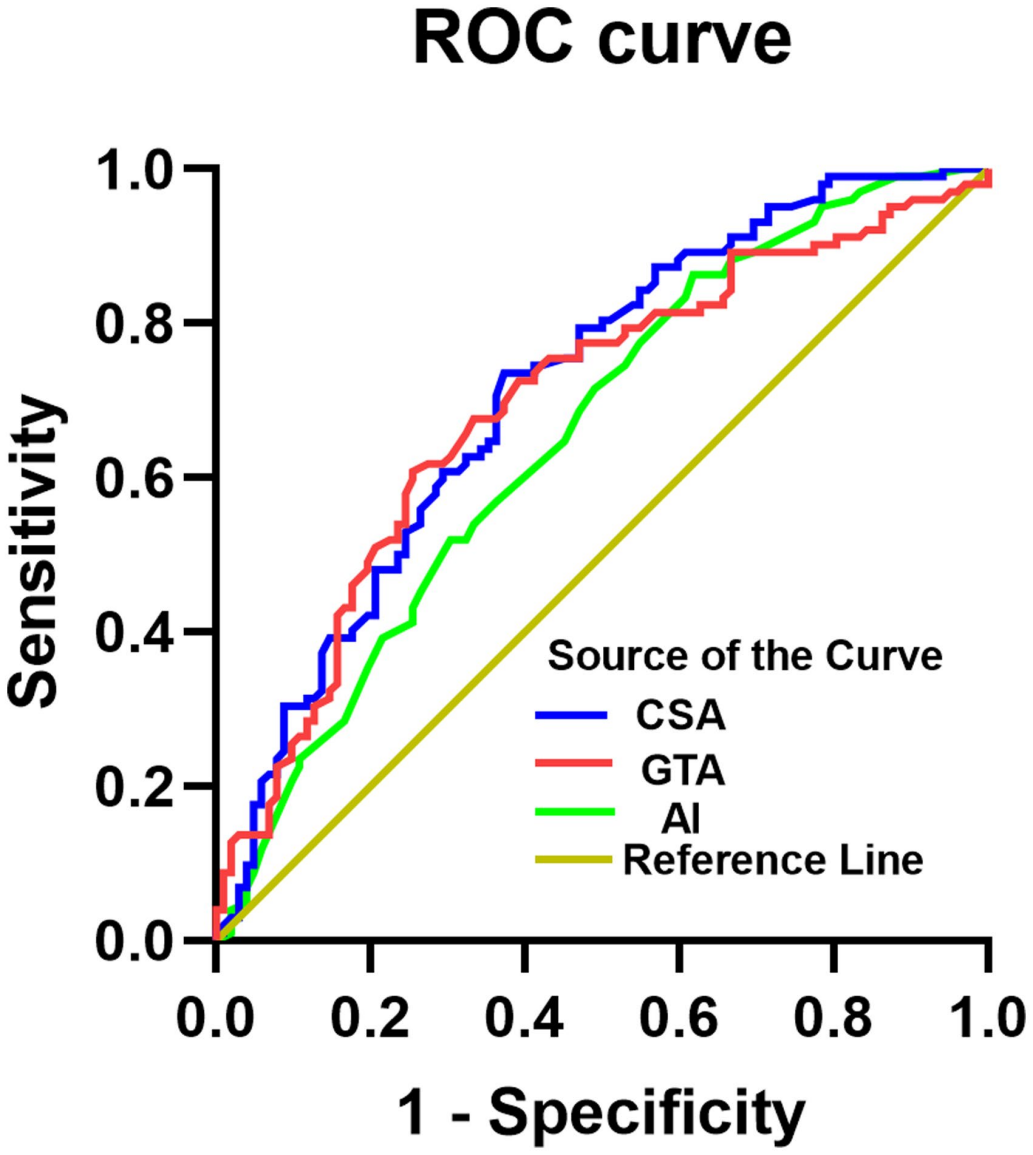
ROC curves for CSA in blue, GTA in red and the AI in green. The reference line is indicated in yellow. ROC, receiver operating characteristic; CSA, critical shoulder angle; GTA, greater tuberosity angle; AI, acromial index.

ROC curve analysis determined cutoff values for discriminating the bursal-sided PTRCTs and control groups for the AI (> 0.635), CSA (> 32.75°), and GTA (> 71.65°). The area under the ROC curve was highest for the CSA [0.714 (95%CI 0.644–0.784) vs. 0.655 (95%CI 0.580–0.729) and 0.695 (95%CI 0.622–0.767) for the AI and GTA, respectively], indicating that the CSA is the most valuable measure for discriminating between the bursal-sided PTRCTs and control groups. The sensitivity of CSA was lower than AI (0.735 vs. 0.863), however, higher than GTA (0.735 vs. 0.608). On the other hand, the specificity of CSA was higher than AI but lower than GTA (0.627 vs. 0.382 vs. 0.745, respectively; Table 3; Figure 5).

**Table 3:** Receiver operating characteristic analysis of AI, CSA, and GTA for bursal-sided partial thickness of rotator cuff tears.

Variable	AUC (95%CI)	SE	Cutoff value(°)	Sensitivity(%)	Specificity(%)	Youden index	*p* value
AI	0.655 (0.580–0.729)	0.038	0.635	0.863	0.382	0.245	< 0.001
CSA	0.714 (0.644–0.784)	0.036	32.75	0.735	0.627	0.362	< 0.001
GTA	0.695 (0.622–0.767)	0.037	71.65	0.608	0.745	0.353	< 0.001

AI, acromial index; CSA, critical shoulder angle; GTA, greater tuberosity angle; AUC, area under the curve; CI, confidence interval; SE, standard error.

### Reliability of radiologic measurements

The ICCs and kappa values of all radiologic measurements were reliable, with intra-observer ICCs of 0.98 for CSA, 0.95 for GTA, 0.91 for AI, with intra-observer kappa values of 0.89 for acromial spur. Additionally. with inter-observer ICCs of 0.96 for CSA, 0.89 for GTA, 0.89 for AI, with intra-observer kappa values of 0.81 for acromial spur. All these radiologic measurements have excellent agreement (*p* < 0.001; Table 4).

**Table 4:** Reliability of radiologic measurements.

Variable	Intra-observer reliability		Inter-observer reliability	
Categorical data	Kappa (95% CI)	*p* value	Kappa (95% CI)	*p* value
Acromion type	0.89 (0.83–0.91)	< 0.001	0.82 (0.75–0.89)	< 0.001
Acromial spur	0.89 (0.78–0.99)	< 0.001	0.81 (0.67–0.95)	< 0.001
Continuous data	ICC (95% CI)	*p* value	ICC (95% CI)	*p* value
CSA	0.98 (0.96–0.99)	< 0.001	0.96 (0.95–0.97)	< 0.001
GTA	0.95 (0.93–0.96)	< 0.001	0.89 (0.85–0.92)	< 0.001
LAA	0.96 (0.94–0.97)	< 0.001	0.94 (0.93–0.96)	< 0.001
β-angle	0.90 (0.85–0.93)	< 0.001	0.86 (0.81–0.89)	< 0.001
AI	0.91 (0.86–0.94)	< 0.001	0.89 (0.86–0.92)	< 0.001
AHD	0.88 (0.84–0.92)	< 0.001	0.82 (0.76–0.86)	< 0.001
AT	0.97 (0.96–0.98)	< 0.001	0.95 (0.93–0.96)	< 0.001
AS	0.96 (0.94–0.97)	< 0.001	0.94 (0.91–0.95)	< 0.001

ICC, intraclass correlation efficient; CI, confidence interval; CSA, critical shoulder angle; GTA, greater tuberosity angle; LAA, lateral acromial angle; AI, acromion index; AHD, acromiohumeral distance; AT, acromial tilt; AS, acromial slope.

## Discussion

Studies have revealed intratendinous tears in 55%, articular-side tears in 27%, and bursal-side tears in only 18% of cadavers with PTRCTs (29, 30). Although bursal-sided tears are less common than intratendinous and articular-sided tears, they are more painful and have a more significant impact on a patient’s quality of life due to the presence of more nerve fibers and blood vessels on the bursal side (29). Hence, studies focusing on bursal-sided PTRCTs are of great significance. The main finding of our study was that acromion spur, GTA, CSA, and AI are independent risk factors for bursal-side PTRCTs and that CSA is a strong predictive factor for bursal-side PTRCTs compared to GTA and AI.

LAA was first described by Banas et al. (19), who reported that a lower LAA was significantly correlated with RCTs. A study by Balke et al. (31) reported similar findings, noting that patients with degenerative RCTs have lower LAA than those with traumatic RCTs. The data of this study further verified this finding and reported that patients with bursal-side tears had a lower mean value of LAA than those with intact rotator cuff tendons. Tetreault et al. (32) found that patients with a lower LAA have a narrower subacromial space. During shoulder abduction, the rotator cuff is susceptible to impingement with the subacromial surface and coracoacromial ligament, resulting in RCTs. Bursal-side PTRCTs are strongly associated with subacromial impingement syndrome (7). Due to its anatomical position, the bursal-side rotator cuff, which makes direct contact with the subacromial surface or coracoacromial ligament during shoulder abduction, is susceptible to abrasion, degeneration, and eventual tears.

The CSA reflects not only the lateral extension of the acromion but also glenoid inclination, integrating both potential risk factors into one radiologic parameter (33). CSA was first studied by Moor et al. (5), who found that patients in the RCT group had a higher CSA than controls (38° vs. 33°, *p* < 0.001) and that patients with CSA > 35° are associated with a high prevalence of RCTs. This result was consistent with other studies conducted by Andrade et al. (16) and Zaid et al. (15). Reasons for analyzing the significant correlation between CSA and RCTs are as follows; on the one hand, the larger the lateral extension of the acromion is, the greater the likelihood for it to lead to RCTs (4); on the other hand, the overload of the tendon caused in part by increased glenoid inclination could contribute to the development of RCTs (34). Our study further confirmed these findings. We found that the mean CSA were significantly higher in the bursal-sided PTRCTs patients than in the controls and that CSA was an independent risk factor for bursal-sided tears (Figure 3). In addition, our analysis showed a moderate correlation between CSA and AI (Figure 4), which is similar to the results of Liu et al. (12) and Heuberer et al. (35).

Similar to CSA, AI can also reflect the lateral extension of the acromion, a factor which was introduced by Nyffeler et al. (4) in 2006. In our study, we found that the mean AI was significantly higher in bursal-side PTRCTs patients than in the controls, and the multivariate analysis demonstrated that a large AI was an independent risk factor for bursal-side PTRCTs. Extrinsic causes, such as subacromial impingement, are common in bursal-side PTRCTs (36). After excluding other potentially relevant influencing factors, we conclude that the AI correlates positively with the lateral extension of the acromion and that a large lateral extension of the acromion predisposes the supraspinatus tendon to degeneration due to its influence on the orientation of the resultant deltoid muscle force vector (4)

Cunningham et al. (20) introduced GTA as a reliable radiographic marker for detecting RCTs. Yoo et al. (37) showed a significant correlation between a larger GTA and RCTs. Seo et al. (38) and Nyffele et al. (39) also expressed the same opinion, with Nyffele et al. (39) noting that bursal-sided tears might be caused by friction and abrasion of the tendon. Our study showed a significantly higher GTA in the bursal-sided PTRCTs group than in the control group, and GTA was an independent risk factor for bursal-sided PTRCTs. Higher GTAs may be correlated with bursal-sided PTRCTs for the following reasons. Because the GTA considers both horizontal and vertical migration of the greater tuberosity of the humerus (20), in patients with a higher GTA, the greater tuberosity is elevated, causing the supraspinatus tendon to stop closer to the subacromial surface. Therefore, during abduction of the shoulder joint, the bursal-sided tendon is more susceptible to damage caused by subacromial impingement, resulting in tendon abrasion and later development of bursal-sided PTRCTs.

For the relationship between β-angle and glenoid inclination, Daggett et al. (34) defined glenoid inclination as β-angle subtracted from 90, with positive values representing superior glenoid inclination and negative values inferior glenoid inclination. This paper also showed that glenoid inclination is significantly increased in patients with massive rotator cuff tears. In addition, Beeler et al. measured the β-angle using X-ray (40) and CT (41) measurements and found that β-angles were significantly smaller in patients with RCTs than in controls. The results of this study are consistent with the above findings. We found that the mean β-angle was significantly smaller in the bursal-sided PTRCTs group than in the control group (71.0° vs. 74.2°, *p* = 0.009), suggesting that patients with a larger uptilting of the scapula were more likely to develop bursal-sided PTRCTs. Possible causes for this result may include the increasing load on the supraspinatus tendon with increased scapular glenoid inclination because consistently overloading the supraspinatus tendon tends to lead to RCTs (42).

The most common classification for acromial morphology is the one by Bigliani et al. (3) describing a flat (type-I), curved (type-II), or hooked (type-III) acromion on outlet-view radiographs. There was no statistically significant difference between the bursal-sided PTRCTs group and the control group in this study, indicating that there was no relationship between acromial morphology and bursal-sided tears. In addition, Akram et al. (43) demonstrated that type II acromion accounted for 56.4% of the cases studied and was the most common acromion type, which is consistent with this study’s findings. We found that the most common acromion type was curved in both groups, with 52.0% in the bursal-sided PTRCTs compared to 49.0% in the control group (Table 2). Similarly, our study found that AT, AS and AHD were not significantly associated with bursal-sided PTRCTs; however, further studies are needed to confirm this conclusion.

An acromial spur is a convex inferior surface of the acromion. Gagey et al. (44), who first described an inferior enthesophyte of the acromion, conducted an MRI scan of 179 shoulders and found that 45.5% of patients with subacromial impingement syndrome had a subacromial enthesophyte, suggesting a strong association between an inferior enthesophyte and subacromial impingement syndrome. A subsequent study by Farley et al. (45) characterized the acromion with an inferior enthesophyte as a type IV acromion based on Bigliani et al. (3). In our study, 15 patients (14.7%) in the bursal-sided PTRCTs group had a convex inferior surface of the acromion, compared to 4 patients (3.9%) in the control group. There were significant differences between the two groups (*p* = 0.014, Table 2), suggesting a significant correlation between acromial spurs and bursal-sided PTRCTs. Further multivariate logistic regression revealed that the presence of an acromial spur was an independent risk factor for bursal-sided PTRCTs.

It is worth noting that some studies have shown a significant association between smoking and RCTs (46–48). A study by Brooks et al. (49) demonstrated that the supraspinatus/infraspinatus tendon was hypovascular in their distal 15 mm. In addition, Mosely et al. (50) showed that Nicotine is a potent vasoconstrictor and decreases oxygen delivery to tissues. Therefore, it is not difficult to conclude that smoking can further compromises the vascular supply to this zone above involved, which leads to an increased risk of RCTs. However, this study found no significant difference between bursal-sided PTRCTs and controls (*p* = 0.079), which may be because all cases included were PTRCTs.

Finally, we confirmed the association between larger CSAs and bursal-sided PTRCTs. Additionally, CSA was the most powerful predictor of bursal-sided PTRCTs, with an area under the ROC curve of 0.714, indicating a probability that at least 71.4% of patients with bursal-sided tears have a larger than normal CSA. Similar results were reported by Moor et al. (33), Pandey et al. (51), and Watanabe et al. (52). While although there was a higher CSA in bursal-sided PTRCTs, the area under the ROC curve showed a low diagnostic value; thus, the use of this parameter to diagnose bursal-sided PTRCTs should proceed with caution. Likewise, a patient’s CSA might have some bearing on any decisions regarding their clinical care.

This investigation has some limitations. First, this is a retrospective study, and further validation of the results by high-quality randomized controlled clinical trials is needed in the future. Second, photographic and measurement errors could exist despite excellent interobserver agreement. Third, potential selection bias may exist owing to strict inclusion criteria. Finally, since MRI or computed tomography was not used, the accuracy of radiographic measurement was not further verified.

## Conclusion

Our study revealed that acromial spur, GTA, CSA, and AI were independent risk factors for bursal-sided PTRCTs. Furthermore, CSA was the most powerful predictor of bursal-sided PTRCTs compared to GTA and AI.

## Data availability statement

The original contributions presented in the study are included in the article/supplementary material, further inquiries can be directed to the corresponding author.

## Ethics statement

The studies involving human participants were reviewed and approved by The Second Affiliated Hospital of Nanchang University Medical Research Ethics Committee. The patients/participants provided their written informed consent to participate in this study.

## Author contributions

JL and SD contributed to the data acquisition. HD, DQ, LL, ML, and JK contributed to data analysis, interpretation, and drafting. JL, ZC, and JT conceived and designed the study. JT were in charge of the conceptualization, supervision, and project administration. All authors read and approved the final manuscript.

## Funding

This work was supported by the Science and Technology Bureau of Nanchang City, Jiangxi Province (2020-NCZDSY-009).

## Conflict of interest

The authors declare that the research was conducted in the absence of any commercial or financial relationships that could be construed as a potential conflict of interest.

## Publisher’s note

All claims expressed in this article are solely those of the authors and do not necessarily represent those of their affiliated organizations, or those of the publisher, the editors and the reviewers. Any product that may be evaluated in this article, or claim that may be made by its manufacturer, is not guaranteed or endorsed by the publisher.
